# Nutritional and Techno-Functional Evaluation of Faba Bean (*Vicia faba* L.) Flour and Protein Concentrate

**DOI:** 10.3390/foods15081350

**Published:** 2026-04-13

**Authors:** Jessica Noelia Perez, María Victoria Salinas, Antonio Francisco Guerrero Conejo, María Cecilia Puppo

**Affiliations:** 1 Center for Research and Development in Food Science and Technology (CIDCA, CCT-CONICET), National University of La Plata, La Plata RA1900, Argentina; jesper@alum.us.es; 2 Higher Polytechnic School, University of Seville, Calle Virgen de África, 7, 41011 Sevilla, Spain

**Keywords:** *Vicia faba* L., faba bean, legume ingredients, hydration property, sustainable protein source, techno-functional

## Abstract

The aim of this work was to evaluate the compositional, functional, antioxidant, thermal, and structural properties of faba bean flour (FBF) and a faba bean protein concentrate (FBC) elaborated by a sustainable dry fractionation method. Proximate composition was determined by analyzing the content of moisture, protein, lipids, ash, total dietary fiber, starch, and available carbohydrates. A methanolic extract was used to analyze total polyphenols and antioxidant capacity using complementary methods. The fatty acid profile was determined by gas chromatography. Techno-functional properties were assayed, determining water-holding capacity (WHC), oil absorption capacity (OAC), and retention capacity of different solvents (SRC), water activity, pH, and titratable acidity. Structural and thermal properties were studied by FTIR and DSC. The dry method produces a concentrate with a low quantity of starch (2.5 vs. 25.6%) and carbohydrates and higher amounts of proteins (61.14 vs. 23.61%). Lipids, mainly mono and polyunsaturated ones, and polyphenols with high antioxidant activity. FBC absorbed a greater proportion of lactic acid, likely due to its higher acidity, and showed higher oil absorption, but retained less water compared to FBF. FTIR and DSC results suggested that the heat-treated proteins (in FBC) exhibited some degree of protein denaturation, unlike the FBF proteins. These findings highlight the potential of dry-fractionated faba bean concentrate as a sustainable and functional food ingredient, particularly for products aimed at improving nutritional quality. Its enhanced antioxidant profile, favorable lipid composition and unique techno-functional properties make it a promising alternative for developing plant-based foods.

## 1. Introduction

Faba beans belong to the Fabaceae family, the Vicia genus and the *Vicia faba* L. species. *Vicia faba* is a temperate legume that adapts well to fertile soils and contributes to soil fertility through symbiotic nitrogen fixation with rhizobia. Due to its low nitrogen fertilizer requirement and its positive effect on soil quality, it is widely used in crop rotations and considered a sustainable agricultural species [1,2]. According to the Food and Agriculture Organization of the United Nations, dry bean seeds are cultivated worldwide, occupying an estimated area of 2.7 million hectares, and a production of more than 6.07 million tons [3]. The consumption of beans has declined in some regions due to food diversification and the emergence of new sources of protein. However, they remain an important food legume in several regions, including China, Egypt, Ethiopia, and some Mediterranean countries [3]. Faba beans are commercially available fresh, dried, canned, or frozen, and are used in the development of various foods and gluten-free alternatives, such as spaghetti, bread, and yogurt [4]. Although it can improve the protein content of formulations, its bitter taste and limited functional characteristics present certain technological challenges [5].

Faba beans are valued for their high protein content, which generally ranges from 20% to 35%. In addition, Faba bean seeds contain high carbohydrate content (51–68%) with starch accounting for the largest portion (approximately 41–58%) [6] and high amounts of dietary fiber with values of 27.5% reported by Feng et al. [7]. Among the soluble sugars, the predominant ones belong to the raffinose family of oligosaccharides, including raffinose, stachyose, and verbascose; particularly, faba beans are rich in stachyose and verbascose [6]. It also contains minerals such as iron, magnesium, zinc, and phosphorus [7].

Bean flour is produced by grinding clean and dried seeds. In contrast, the protein concentrate is obtained by selectively removing non-protein components, mainly starch and fiber, resulting in a protein content of between 60% and 90%. These protein concentrates hold significant potential in the food industry as substitutes for animal proteins or as techno-functional ingredients. When incorporated into food and non-food products, plant-based proteins contribute to valuable functional properties, including solubility, viscosity, foaming, emulsification, gelling, and the ability to bind water and oil [5,8,9]. In addition, proteins play a crucial role in determining the sensory, physicochemical, and organoleptic properties of food products, including texture, flavor, and color [10]. There are various methods available for obtaining legume protein concentrates, including wet extraction, ultrafiltration, and dry fractionation. Although wet processes are effective, they require large amounts of water and chemicals, raising environmental concerns. In contrast, dry fractionation, which involves milling followed by air classification, is considered a more sustainable approach as it does not require solvents and involves lower energy consumption. This method separates particles according to their size and density, producing a protein-rich and a starch-rich fraction. It preserves the native structure and functional properties of proteins more effectively than conventional wet extraction methods. Furthermore, faba bean protein concentrates obtained by dry fractionation have a lower environmental impact, partly due to the reduced processing requirements [11,12,13]. Beans contain four types of protein, the majority of which are globulins (70–78%), followed by albumins (10–20%), glutelins (around 15%), and prolamins (less than 5%) [14]. Based on their sedimentation coefficients (S), faba bean globulins are classified into two main types: legumin (11 S, 300–400 kDa) and vicilin (7 S, ~180 kDa). The predominant protein band observed in faba bean by SDS-PAGE at 63 kDa corresponds to the 11 S legumin fraction [15], composed of subunits of 41 and 22 kDa linked by disulfide bonds [16]. These authors also reported that the 7 S vicilin fraction ranges between 29 and 52 kDa. Despite increasing interest in faba bean protein ingredients, there is limited information available on the detailed composition, structure, and techno-functional properties of protein concentrates obtained by dry fractionation. Most previous studies have focused on wet extraction methods or whole flour, and there is little comparative data between flour and dry-fractionated concentrates. This study hypothesized that dry fractionation of faba bean flour would produce a protein-rich fraction with a modified composition and enhanced techno-functional properties compared to the original flour. This would increase its potential as a sustainable, plant-based food ingredient. Therefore, the objective of this study was to analyze the proximate composition of faba bean flour (FBF) and faba bean concentrate (FBC) obtained through dry fractionation and to compare their nutritional characteristics, polyphenol content, and antioxidant capacity. Additionally, the technological properties of the samples were evaluated, including their solvent retention capacity, water-holding capacity (WHC), oil absorption capacity (OAC), water activity, and acidity. Structural and thermal properties were also assessed using Fourier-transform infrared spectroscopy (FTIR) and differential scanning calorimetry (DSC).

## 2. Materials and Methods

### 2.1. Materials

#### 2.1.1. Faba Bean Flour (FBF)

Dried faba bean seeds, donated by the organization Red Puna y Quebrada from northern Argentina (harvested in 2021), were used. The whole seeds, including the husks, were pre-ground in a manual grain mill before being passed through a Perten Instrument 3170 Mill Feeder (manufactured in Winnipeg, MB, Canada) to produce FBF with a particle size of 500 µm. The flour was stored in a closed plastic container at −20 °C until use.

#### 2.1.2. Faba Bean Concentrate (FBC)

The Herba PROFB65 bean protein concentrate was supplied by Herba Ingredients of San José de la Rinconada, Seville, Spain. This product was obtained using the dry fractionation method. The product contains approximately 59–70% protein (N × 6.25), moisture < 13.5%, and a particle size distribution d90 of 10–50 µm. The concentrate is supplied as a fine powder and stored under dry and cool conditions in unopened packaging.

### 2.2. Methods

#### 2.2.1. Chemical Composition

The composition of faba bean flour and concentrate was determined according to AOAC methods [17]. Moisture was measured by oven drying at 105 °C (AOAC 925.10), ash in a muffle at 550 °C (AOAC 923.03), protein by the Kjeldahl method (AOAC 979.09, N × 6.25), and lipids by Soxhlet extraction (AOAC 920.39). Total dietary fiber (TDF) by the enzymatic–gravimetric method (AOAC 991.43) and total starch (AOAC 996.11) using the Megazime kits (Megazime, Wicklow, Ireland).

The content of available carbohydrates (AC) other than starch was calculated by difference (AC = 100 − moisture − protein − lipids − TDF − ash − total − starch). Assays were performed in triplicate. All results were expressed in g per 100 g of flour.

#### 2.2.2. Total Polyphenols Content: Polyphenol Extraction

Extracts from FBF and FBC were prepared using a modified method used by Avallone et al. [18]. Flour (0.5 g) was extracted three times with 3, 3, and 2 mL of methanol: water mixture (80:20, *v*/*v*), with stirring for 2 h at 25 °C and 750 rpm in a Thermomixer (DLAB Scientific, model HCM100-Pro, Beijing, China). The supernatants were combined and centrifuged at 1333× *g* for 10 min. The extracts were stored at −16 °C until analysis. Total phenolic content and antioxidant activity were assessed in triplicate.

##### Determination of Total Phenolic Content

Total polyphenols were determined by the Folin–Ciocalteu method with some modifications [19]. The extract (75 μL) was mixed with distilled water (1100 μL) and FC reagent (25 μL). After 2 min, 100 μL of a 20% *w*/*v* Na2CO3 (in 0.1 N NaOH) was added and allowed to stand in the dark for 60 min. Absorbance was measured at 750 nm using gallic acid as a standard in a microplate reader (BioTek 800 TC, Shoreline, WA, USA). The calibration curve was prepared using gallic acid in the concentration range of 0–0.256 mg/mL. The regression equation obtained was y = 0.2268x − 0.0003 (R^2^ = 0.99). Total polyphenols were expressed as mg gallic acid per gram of sample on a dry weight basis (mg GA/g).

##### Determination of Antioxidant Capacity: Ferric Ion Reducing Antioxidant Power Assay (FRAP)

The FRAP assay was performed with some modifications to the original technique [20]. Extract (100 μL) was mixed with 900 μL of FRAP reagent, and absorbance was measured at 593 nm after 30 min. A calibration curve (0–96 μg/mL) was prepared with Trolox (6-hydroxy-2,5,7,8-tetramethylchroman-2-carboxylic acid). The calibration curve was prepared using Trolox (6-hydroxy-2,5,7,8-tetramethylchroman-2-carboxylic acid) in the concentration range of 0–96 µg/mL. The regression equation obtained was y = 0.0095x + 0.1629 (R^2^ = 0.99). Results were expressed as μmol Trolox per g of sample on a dry weight basis (µmol T/g).

##### Free Radical Scavenger Activity on 2,2-Diphenyl-1-picrylhydrazyl (DPPH)

The evaluation of radical scavenging activity against DPPH was performed according to Teixeira et al. [21], with modifications. Extracts (50 μL) were mixed with 950 μL of 35 mg/L DPPH ethanolic solution. After 60 min in the dark, absorbance was read at 515 nm. Trolox (0–320 μg/mL) was used for the standard curve. The calibration curve was prepared using Trolox in the concentration range of 0–320 µg/mL. The regression equation obtained was y = −0.0012x + 0.5717 (R^2^ = 0.99). Results were expressed as μmol Trolox per g of sample on a dry weight basis (µmol T/g).

##### Free Radical 2,2′-Azino-bis-(3-ethylbenzothiazoline-6-sulfonic Acid) (ABTS+)

The reaction between 7 mM ABTS and 2.45 mM potassium persulfate for 12 h in the dark was used to generate the ABTS^+^ radical. The solution was diluted to achieve an absorbance of 0.70 ± 0.02 at 734 nm. For the assay, 20 µL of the diluted extract was mixed with 1000 µL of ABTS^+^ solution, and absorbance was measured at 734 nm after 20 min [22]. The calibration curve was prepared using Trolox in the concentration range of 0–64 µg/mL. The regression equation obtained was y = −0.0065x + 0.5986 (R^2^ = 0.99). Results were expressed as μmol Trolox per g of sample on a dry weight basis (µmol T/g).

#### 2.2.3. Fatty Acids Profile of Faba Beans Flour and Concentrate

The fatty acid (FA) profile was determined by gas chromatography [23]. Lipids were extracted using the Folch method, derivatized with methanolic HCl solution, heated in boiling water, and treated with hexane and centrifuged (1333× *g* for 15 min). The organic phase containing the fatty acid methyl esters (FAMEs) was filtered and subsequently injected into an Agilent Technologies 7890A gas chromatograph (Agilent Technologies; Santa Clara, CA, USA) using a Supelco DB 23 capillary column and a flame ionization detector (FID). A FAME Mix (Sigma-Aldrich, St. Louis, MO, USA) was used as a reference standard for fatty acid identification. Peak areas were analyzed using PeakFit software (version 4.12 for Windows, SPSS Inc., Chicago, IL, USA). Fatty acid analyses were performed in duplicate.

Atherogenicity index (AI), thrombogenicity index (TI), and the hypocholesterolemic/hypercholesterolemic ratio (h/H) were calculated using the percentage of FA according to the following equations:

(1) AI = [C12:0 + (4 × C14:0) + C16:0]/(MUFA + n3 PUFA + n6 PUFA)

(2) TI = (C14:0 + C16:0 + C18:0)/[(0.5 × MUFA) + (0.5 × n6 PUFA) + (3 × n3 PUFA) + (n3 PUFA)/(n6 PUFA)]

(3) h/H = (C18:1+ C18:2 LA + C18:3 ALA)/(C12:0 + C14:0 + C16:0)

#### 2.2.4. Techno-Functional Characteristics of Faba Beans Flour and Concentrate

Techno-functional properties of faba bean flour (FBF) and protein concentrate (FBC) were assessed by different methods by measuring water-holding capacity (WHC), oil absorption capacity (OAC), and solvent retention capacity (SRC) using [17]. Additionally, water activity at 25 °C in Meter AquaLab series 4 TEV equipment (Decagon Devices Inc., Pullman, WA, USA), and titratable acidity [17] were studied. Assays were performed in triplicate.

#### 2.2.5. Structural and Thermal Properties of the Sample Suspension: Protein and Starch Analysis by Fourier Transform Infrared Spectroscopy (FTIR)

An Attenuated Total Reflectance Fourier Transform Infrared spectrophotometer (ATR-FTIR) was used to obtain the spectra (*n* = 10) according to Guardianelli et al. [23]. The samples were previously reduced in particle size in a curing mortar and then placed in a Thermo Nicolet iS10 ATR-FTIR spectrophotometer (Thermo Scientific, Waltham, MA, USA). The molecular spectral data of flour and concentrate of faba bean were collected and corrected with the water background, and further analyzed with OMNIC software (version 8.3, Thermo Scientific, Waltham, MA, USA). Thirty-two scans were taken with a spectral resolution of 4 cm^−1^ in the range 4000–500 cm^−1^. The average value of the spectra was determined with baseline correction between 1720 and 1585 cm^−1^ and 935–1103 cm^−1^ for the Amide I and starch bands, respectively. The second inverse derivative was performed on them to estimate the position and relative proportion (%) of the components of Amide I (1580–1710 cm^−1^) associated with the secondary structure of proteins, and bands between (940–1105 cm^−1^) associated with starch. Subsequently, the protein and the starch bands were fitted to a Gaussian-Lorentzian type profile.

#####  Differential Scanning Calorimetry (DSC)

The thermal properties of FBF and FBC were determined in aqueous suspensions (20% *w*/*v*) using a Q100 instrument (TA Instruments, New Castle, DE, USA) [23]. About 6 mg of suspension was placed in aluminum capsules that were hermetically and manually sealed using a TA manual press supplied with the DSC equipment to ensure hermetic closure prior to analysis. Sealed capsules were then subjected to one heating cycle, heating from 20 °C to 120 °C at a rate of 10 °C/min. Protein denaturation and/or starch gelatinization were characterized by different temperatures: onset (T0), peak, and end (Tf). The enthalpy change (ΔH) associated with the endothermic process was determined between T0 and Tf. Assays were performed in duplicate.

#### 2.2.6. Statistical Analysis

Data was analyzed using InfoStat [24] software (version 2024). Normality and homogeneity of variance assumptions were previously verified using Shapiro–Wilk and Levene’s tests, respectively. A one-way analysis of variance (ANOVA) was employed, followed by the least squares difference (LSD) test for multiple comparisons (*p* < 0.05). The specific number of replicates (*n*) for each assay is indicated in its respective methodological description above. All values are expressed as means ± standard deviation (SD).

## 3. Results and Discussion

### 3.1. Composition and Nutritional Characterization of Faba Bean Flour and Concentrate

Faba Bean Concentrate (FBC) exhibited a higher moisture content than FBF (Table 1), which may be attributed to the presence of hygroscopic components. In addition, FBC compared to FBC contained approximately three times more protein than FBF and approximately twice the lipids and ash contents. In contrast, FBF presented higher levels of carbohydrates, particularly total dietary fiber (21.05% vs. 15.32%), total starch (25.6% vs. 2.5%), and available carbohydrates (18.0% vs. 0.78%). The higher protein content achieved through dry fractionation is because of separating the different fractions based on their densities during processing, leading to the mechanical removal of particles of high fiber, especially with significant amounts of starch and available carbohydrates. High protein contents (20–41%) were reported for different varieties and sources of faba bean flour and concentrates [25]. A study conducted in 2022 reported similar average values for proteins, lipids, and minerals in faba beans, but lower values for total dietary fiber, although higher values for starch [26].

### 3.2. Total Phenolic Compounds (TPC) and Antioxidant Capacity

**Figure 1:**
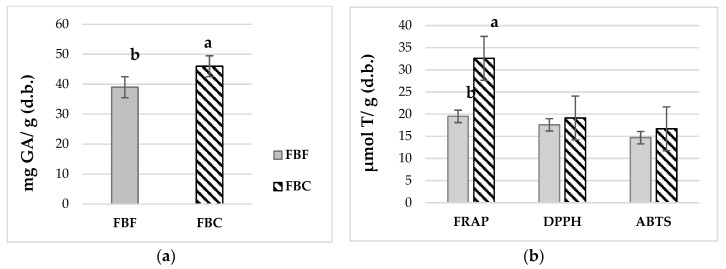
(**a**) Total polyphenols content in Faba Bean Flour (FBF) and Faba Bean Concentrate (FBC), and (**b**) antioxidant capacity assessed by FRAP, DPPH, and ABTS assays. Values (dry weight basis, d.b.) are expressed as mean ± SD (*n* = 3). Different letters indicate statistically significant differences (*p* < 0.05).

The content of total soluble polyphenols was significantly higher in FBC than in FBF, with values of 39 mg GA/g of FBF and 47 mg GA/g of FBC, respectively (Figure 1a). The higher antioxidant values observed in FBC could be partially associated with its higher moisture content and compositional differences, which may affect the extraction yield of phenolic compounds. The distribution of phenolic compounds varies considerably among the different parts of the faba bean. The highest concentrations are found in the seed coats. A previous study analyzed the TPC of various bean fractions (whole pod, pod cover, whole seed, seed cover, and cotyledon), finding a value of 36.35 mg GA/g (d.b.) for whole seeds [27].

This value was like that obtained by FBF. Figure 1b shows the antioxidant activity measured using three complementary methods. The FBC exhibited higher antioxidant activity by FRAP than the FBF, while no significant differences were observed using the DPPH and ABTS methods. In all the techniques tested, it was found that the antioxidant capacity was higher in FBC than in FBF. Other authors previously studied the antioxidant capacity of faba beans. Faba Beans from Peru, ground and extracted with a solution of methanol, water, and acetic acid, obtained higher antioxidant activity values for ABTS (38 µmol T/g of dry bean) and lower values for DPPH (6.6 µmol T/g of dry bean) [28]. A recent study examined the polyphenol content and antioxidant activity of three varieties of Peruvian beans. The polyphenols obtained (0.79–1.25 mg GA/g) and antioxidant activity measured by DPPH (4.68–5.17 µmol T/g) were lower than those obtained in this FBF trial. On the other hand, similar antioxidant potential values were observed using the ABTS assay (16.60–21.01 µmol T/g) [29]. Previous studies conducted on Australian faba beans obtained similar values for FRAP (18.2 μmol Fe^+2^/g d.b.), but higher values for DPPH (53.4 μmol T/g d.b.) [30]. In 2012, an isolate of faba beans was characterized and found to have an antioxidant activity of 45.7 (μmoles T/μg of isolate) as evaluated by the ABTS method. However, no other data were found on the polyphenol content and antioxidant activity of protein concentrates or isolates derived from faba bean flour using other techniques [31].

### 3.3. Fatty Acid Profile

The fatty acid (FA) profile influences the nutritional quality of a food ingredient, as the presence of unsaturated FA is a requirement of a healthy diet. The main FAs found in FBF and FBC were palmitic (16:0), stearic (18:0), oleic (18:1), linoleic (18:2), and linolenic (18:3) acids. All these FAs represent 97.2% and 98.7% of the total FAs present in the lipids of FBF and FBC, respectively (Table 2). A similar profile was obtained for the whole faba bean [32]. Analyzing the profile, linoleic acid was the FA present in high amounts in both faba bean samples, and others were identified in very low proportions (<1%) as margaric (17:0), arachidic (20:0), and godonic (20:1) acids (Table 2). The protein concentrate presented significantly lower amounts of saturated FA and higher amounts of total polyunsaturated FA. In addition, FBC exhibited lower values of atherogenicity (AI) and thrombogenicity (TI) indexes, along with a higher hypocholesterolemic/hypercholesterolemic ratio (h/H). Lipid profiles characterized by low AI and TI values, high h/H ratios, and a ω6:ω3 ratio close to 5:1 are considered desirable for maintaining cardiovascular health and supporting a balanced diet.

### 3.4. Techno-Functional Properties of Faba Bean Flour and Concentrate

The techno-functional properties evaluated, including solvent retention capacity (SRC), water-holding capacity (WHC), oil absorption capacity (OAC), water activity (aw), and acidity, are shown in Table 3. Oil absorption capacity (OAC) represents the amount of oil that can be absorbed by a matrix and involves the chemical union between the hydrophobic head of the lipid fraction and the non-polar residues of proteins. When a powder is subject to an excess of water, the amount of water that remains in the sample after compression or centrifugation is evaluated through the water-holding capacity (WHC). Both parameters are directly influenced by the amount and nature of proteins present in flour, and proteins with high values of these parameters are important in several food systems such as meat substitutes, dairy analogs, and baked products [33]. FBC presented a higher OAC than FBF, whereas the opposite behavior was observed for WHC (Table 3). This difference may be attributed to differences in the contents of proteins and carbohydrates of the samples (Table 1). In FBC, the majority component is proteins (61.14%) that contain a high proportion of hydrophobic amino acids responsible for the non-polar interactions with the lipids. Although FBF has a high protein contribution (23.1%), it presents a higher proportion of starch (25.6%) than FBC, a factor that contributes even more to water retention, giving higher WHC values in FBF than in FBC. A study of the technical properties of various legumes, including faba bean flour and faba bean concentrate, found the same trend as in the trial conducted. For the flour, the WHC was higher than the OHC (2.8 vs. 2.5 g/g sample), while for the concentrate, the OHC was higher than the WHC (2.9 vs. 2.6 g/g sample, respectively) [34].

Solvent retention capacity (SRC) of the powders was studied using water and different solutions; values are shown in Table 3. Distilled water interacts globally with all components of the samples; as the chemical composition of both FBF and FBC are different, the sample with the high number of hydrophilic compounds such as proteins and soluble carbohydrates, among others, should present high values of SRC-Distilled Water as was the case of FBF, with values of this parameter almost three times higher than that of FBC. The SRC-Sucrose is related to the pentosan content [35] and to the availability of carbohydrates and fiber; faba beans contain high amounts of starch, and in the testa tissue, there is approximately 35% cellulose and 65% hemicellulose, which form part of the fiber [36]. This is consistent with the higher sucrose retention observed in FBF compared to FBC, likely due to its higher fiber content. During milling, the testa remains in FBF, whereas in FBC, the dry fractionation process removes most of the fiber and starch. The sodium carbonate SRC is associated with the presence of pentosans and the amount of damaged starch in the sample [37]. High values of this parameter are attributed to the easier release of amylopectin due to the damage [38]. Therefore, higher values of this parameter are expected for FBF compared to FBC (Table 3), because in the flour production process, successive milling steps, where mechanical forces can damage starch granules. The SRC-Lactic Acid value of FBC was higher than that of FBF, which is attributed to the higher protein content in FBC that interacts with lactic acid. This SRC is associated with the formation and strength of the protein network. The stabilization of this protein structure is enhanced by the presence of soluble polymeric carbohydrates, such as pentosans, which promote protein–protein interactions and the formation of protein polymers [39]. No data on SRC values of faba bean flours or their derivatives have been reported at present. However, the SRC behavior of wheat flours of different varieties [40] and mixtures of wheat flour and vegetable powder was studied [41]. This latter study showed that the mixture containing the vegetable powder with the lowest fiber content (tomato) had the lowest SRC values in all the solvents tested.

Water activity (aw) is a parameter related to the stability of a food, associated with the shelf-life. Values below 0.75 are considered microbiologically safe [42]. Both samples exhibited aw values below this threshold: 0.418 and 0.635, for FBF and FBC, respectively (Table 3).

Finally, Table 3 shows that FBC presented significantly higher acidity than FBF (3.09 vs. 0.89 g malic acid/100 g sample). This increase is likely due to the higher levels of acidic amino acids associated with the elevated protein levels in FBC. The acidity of FBC followed the same trend as the protein content, being approximately three times higher.

### 3.5. Structural and Thermal Properties of Faba Bean Flour and Concentrate

**Figure 2:**
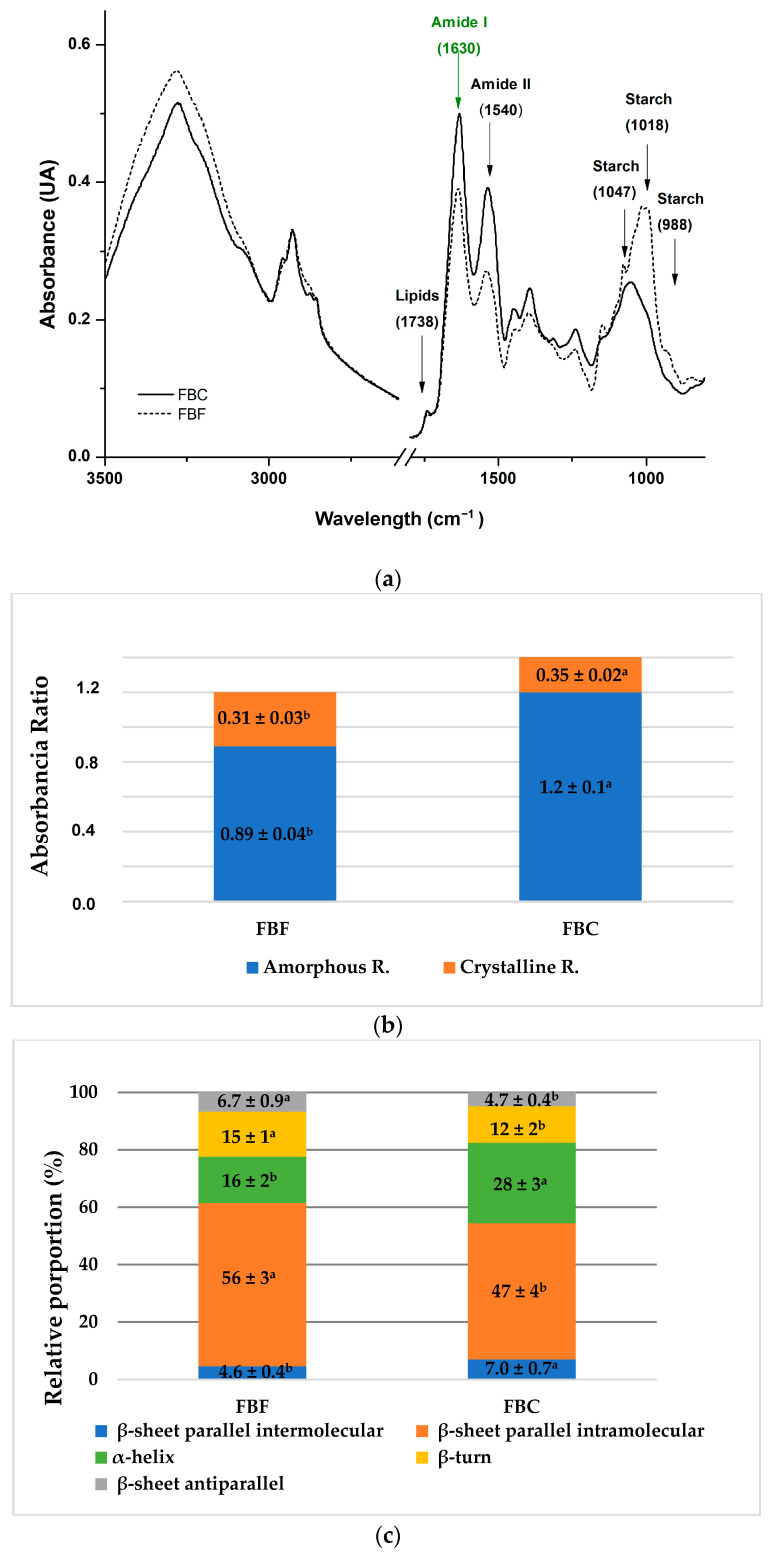
(**a**) FTIR Spectra of Faba Bean Flour (FBF—dotted line) and Faba Bean Concentrate (FBC—entire line). (**b**) Amorphous ratio (1018 cm^−1^/988 cm^−1^) and crystallinity ratio (1047 cm^−1^/988 cm^−1^) for FBF and FBC. (**c**) Secondary structures of proteins analyzed by the Amide I band were expressed as a relative proportion (%) in FBF and FBC. Different letters indicate statistically significant differences (*p* < 0.05).

Figure 2a shows the ATR-FTIR spectra of FBF and FBC samples. Bands corresponding to the major functional groups of lipids, proteins, and carbohydrates (mainly starch) can be observed. For both samples, FBF and FBC, characteristic peaks were identified at different wavenumbers: associated with lipids (1738 cm^−1^), proteins (Amide I: 1630 cm^−1^ and Amide II: 1540 cm^−1^), and starch (1047, 1018, and 988 cm^−1^). Notably, a shift to lower wavenumbers was observed in the starch-associated peaks of the FBC sample, suggesting structural modifications in starch components during the dry fractionation process.

In the lipid region, a peak was observed around 1738 cm^−1^, corresponding to the C=O stretching vibrations of lipid esters [43]. Similarly, a peak was identified near 1700 cm^−1^ in the FTIR spectra of white bean, chickpea, pea, and lentil flours [44]. A peak at 1750 cm^−1^ in a sample of bean pods (*Phaseolus vulgaris*) was attributed to esterified and free carboxyl groups [45].

Three peaks belonging to total carbohydrates, mainly starch, were observed at 988, 1018, and 1045 cm^−1^ for FBF, and 1000, 1047, and 1074 cm^−1^ for FBC. These peaks fall within the 900–1300 cm^−1^ region, associated with C–O and C–C stretching vibrations related to changes in polymer conformation [46]. Previous studies found peaks between 858.84 and 575.34 cm^−1^, attributed to C-O vibrations in ester groups within the C-O-C structure of the glycosidic ring of starch granules in bean flour [47]. In the present study, FBF showed higher peak intensities, which is consistent with its higher carbohydrate content compared to FBC (Table 1).

An attempt at an analysis of the crystalline/amorphous balance of the starch present in both samples was performed by FTIR, analyzing the intensity of the different bands associated with starch structures. The structural properties of starch (amorphous and crystalline regions) were studied using deconvolution analysis, and a Gaussian-Lorentzian mathematical fitting was performed. Specific peaks were identified, and the proportion of crystalline structures ordered relative to the amorphous material in the starches was quantified [46]. The 1018/988 and 1047/1000 peak ratios correlate with the amorphous state of the starch structure, while the 1045/1018 and 1074/1047 ratios reflect the degree of crystalline state of this macromolecule [46,48]. Figure 2b shows that in both FBF and FBC, the amorphous state of starch predominates, as evidenced by both ratios.

Secondary structure of faba bean proteins (Amide I) of FBF and FBC was studied by ATR-FTIR (Figure 2a,c). Two peaks were observed for Amide I (1630 cm^−1^) and Amide II (1540 cm^−1^) structures. The intensity of both bands for FBC was higher with respect to that for FBF, related to the higher content of proteins (Table 1) observed for the concentrate. Previous studies demonstrated that the main amide I bond was the C=O stretch and that its absorption level is modulated by the secondary structure of the protein [49,50].

Deconvolution of the Amide I band was performed on all analyzed spectra. In both cases, five Gaussian-Lorenzian bands belonging to α-helix, intermolecular and intramolecular parallel β-sheet, antiparallel β-sheet, and β-turn structures were obtained. The percentage of these structures is shown in Figure 2c. FBC protein presents a higher amount of α-helix than the FBF protein. Since the α-helix is a more ordered structure, the results suggest that the protein in FBC is in a more native state than in FBF; furthermore, a lower number of β-turns will be observed, relative to the protein in a more disordered state. Despite this, in both samples, the main secondary structure found was β-sheet in all forms, with respect to α-helix and β-turn. These results are consistent with those previously reported by other authors for faba bean proteins [51] and other legumes such as beans, peas, and lentils [52,53], where the proportion of folded β-sheet structures was significantly higher than that of α-helices.

On the other hand, a higher proportion of intramolecular β-parallel sheet structures, compared to intermolecular β-parallel sheets and β-turns in quinoa flours, suggests a greater degree of protein unfolding [23].

**Figure 3:**
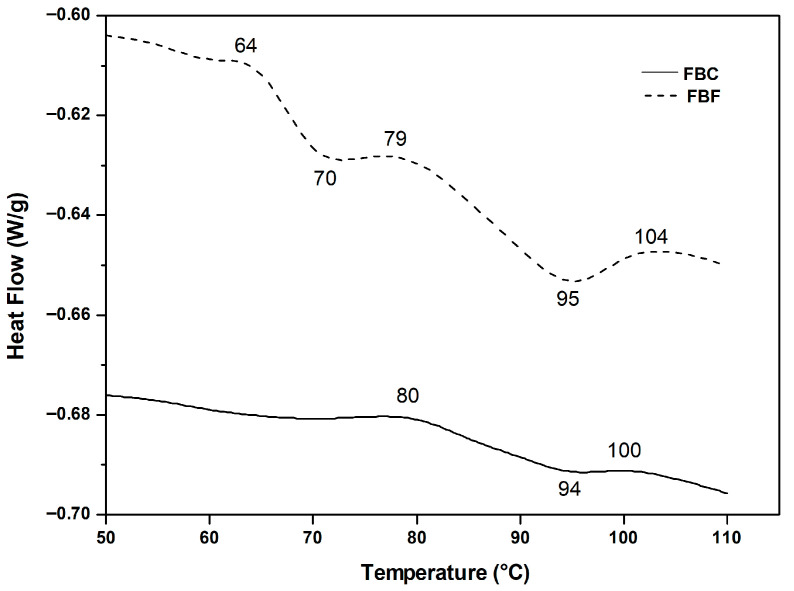
Thermogram obtained by Differential Scanning Calorimetry (DSC) of 20% (*w*/*v*) suspensions of Faba Bean Flour (FBF) and Faba Bean Concentrate (FBC). Different letters indicate statistically significant differences (*p* < 0.05).

Another important parameter that determines the applicability of each sample in plant-based food formulations is its thermal behavior. Figure 3 shows the thermograms obtained by Differential Scanning Calorimetry (DSC) of aqueous dispersions (20% *w*/*w*) of FBF and FBC.

The FBF presented two endothermic peaks at 70 °C and 95 °C, the first of which could be attributed to starch gelatinization, which is consistent with studies previously [54], who found gelatinization temperatures between 61.1 and 72.4 °C with an enthalpy in the range of 1.76–2.27 cal/g. This is because this component is present in large quantities in bean flour (25.6%, Table 1). On the other hand, the second peak at 95 °C would be associated mainly with protein denaturation. In the case of FBC, only one endotherm peak was observed at 94 °C, suggesting that the dry fractionation method effectively removed the starch fraction typically responsible for the lower temperature endotherm around 70 °C due to less content of starch in the concentrate sample (Table 1). This finding is consistent with the denaturation temperature obtained (89.8 °C) in a protein fractionation after applying a combined treatment of salt precipitation and isoelectric precipitation [55]. Years later, a single endothermic peak at 94 °C with an enthalpy change (ΔH) of 2.74 cal/g was reported in a bean protein isolate obtained by dry fractionation [56]. However, it is also known that the denaturation temperatures of the 7S and 11S globulin fractions in beans differ, occurring at 83.8 °C and 95.4 °C, respectively [57].

## 4. Conclusions

This study demonstrates that dry fractionation of faba bean flour (FBF) is effective and allows for the production of faba bean protein concentrate (FBC) with different nutritional, bioactive, and techno-functional profiles. FBC has a significantly higher protein concentration (more than three times that of FBF) and higher levels of lipids, ash, and polyunsaturated fatty acids, while FBF retains more dietary fiber, starch, and available carbohydrates.

The antioxidant capacity and polyphenol content are significantly higher in FBC, highlighting its potential as a functional ingredient. In terms of techno-functional properties, FBC offers greater oil absorption (OAC) due to its protein content, with probably a higher proportion of hydrophobic amino acids. On the other hand, FBF exhibits greater water retention capacity (WHC), as well as better sucrose and sodium carbonate SRC retention, due to its higher fiber and starch content.

Structural analysis reveals greater intensity of Amide I in FBC that presented a higher amount of the ordered α-helix structure than FBF, indicating a molecular organization leading to more stable proteins. FTIR also shows a greater presence of amorphous starch in both materials. Thermally, starch gelatinization and protein denaturation are associated with two endothermic peaks in FBF, while FBC shows a single peak at 94 °C, consistent with protein denaturation and starch elimination.

In summary, the characteristics analyzed indicate that FBC and FBF are attractive ingredients for use in various food matrices, seeking to increase nutritional value and optimize technology. FBC offers high potential for the development of vegetarian protein products and, thanks to its high OAC, as a functionality food enhancer in acidic environments, with good thermal stability and with high bioactivity components (polyphenols). FBF, on the other hand, stands out for its high water-holding capacity (WHC) and good neutral solvent retention, due to its high amorphous starch matrix, making it ideal as a structural ingredient for improving texture and providing fiber. Together, they provide optimal solutions for different requirements in the food industry.

## Figures and Tables

**Table 1:** Proximal composition (*n* = 3) of faba bean flour (FBF) and faba bean concentrate (FBC).

Component (%)	FBF	FBC	*p*-Value
Protein	23.61 ± 0.04 ^b^	61.14 ± 0.07 ^a^	0.0001
Lipids	1.13 ± 0.03 ^b^	2.09 ± 0.05 ^a^	0.0021
Total Dietary Fiber (TDF)	21.05 ± 0.03 ^a^	15.32 ± 0.40 ^b^	0.0024
Ash	3.04 ± 0.03 ^b^	6.20 ± 0.02 ^a^	0.0001
Moisture	7.58 ± 0.10 ^b^	11.97 ± 0.03 ^a^	0.0001
Total starch	25.6 ± 2.0 ^a^	2.5 ± 0.2 ^b^	0.0001
Available carbohydrates	18.0 ± 2.0 ^a^	0.78 ± 0.4 ^b^	0.0001

Different letters in the same row indicate statistically significant differences (*p* < 0.05).

**Table 2:** Fatty acid profile (*n* = 2) of faba bean flour (FBF) and faba bean concentrate (FBC).

Fatty Acids (g of FA/100 g of Lipids)	FBF	FBC	*p*-Value
Palmitic acid (16:0)	17.9 ± 0.4 ^a^	15.53 ± 0.03 ^b^	0.0157
Margaric acid (17:0)	0.32 ± 0.06 ^a^	0.16 ± 0.01 ^a^	0.0782
Stearic acid (18:0)	2.6 ± 0.1 ^a^	1.75 ± 0.04 ^b^	0.0087
Arachidic acid (20:0)	0.91 ± 0.07 ^a^	0.73 ± 0.01 ^a^	0.0665
Total Saturated	21.73 ± 0.1 ^a^	18.17 ± 0.04 ^b^	0.0013
Oleic acid (18:1-ω9)	33.5 ± 1.2 ^a^	27.52 ± 0.04 ^b^	0.0193
Godonic acid (20:1-ω9)	0.42 ± 0.04 ^a^	0.42 ± 0.02 ^a^	0.999
Total Monounsaturated	33.92 ± 1.2 ^a^	27.94 ± 0. 03 ^b^	0.0135
Linoleic acid (18:2-ω6)	41 ± 1 ^b^	51.04 ± 0.02 ^a^	0.1818
Linolenic acid (18:3-ω3)	2.24 ± 0.01 ^b^	2.87 ± 0.06 ^a^	0.0040
Total Polyunsaturated	43.24 ± 1 ^b^	53.93 ± 0.06 ^a^	0.006
ω6/ω3 (−)	18.30	17.78	
AI (−)	0.232	0.189	
TI (−)	0.463	0.359	
h/H (−)	4.287	5.243	

Different letters in the same row indicate statistically significant differences (*p* < 0.05).

**Table 3:** Techno-functional properties (*n* = 3) of faba bean flour (FBF) and faba bean concentrate (FBC). Oil-holding capacity (OAC), water-holding capacity (WHC), and solvent retention capacity (SRC).

Techno-Functional Properties	FBF	FBC	*p*-Value
OAC (g of oil/g)	0.904 ± 0.007 ^b^	1.44 ± 0.07 ^a^	0.0001
WHC (g of water/g)	1.9696 ± 0.0008 ^a^	1.24 ± 0.02 ^b^	0.001
SRC-Distilled water (%)	132 ± 1 ^a^	51 ± 1 ^b^	0.0001
SRC-Sucrose (%)	127 ± 3 ^a^	100 ± 5 ^b^	0.0004
SRC-Sodium carbonate (%)	101.9 ± 0.3 ^a^	82 ± 2 ^b^	0.0001
SRC-Lactic acid (%)	98 ± 2 ^b^	133 ± 1 ^a^	0.0001
Water activity (−)	0.418 ± 0.002 ^b^	0.635 ± 0.009 ^a^	0.0001
Titratable acidity (g malic acid/100 g)	0.89 ± 0.07 ^b^	3.09 ± 0.07 ^a^	0.001

Expressed as g water/oil/solvent per 100 g of sample. Different letters in the same row indicate statistically significant differences (*p* < 0.05).

## Data Availability

The original contributions presented in this study are included in the article. Further inquiries can be directed to the corresponding author.

## Abbreviations

The following abbreviations are used in this manuscript:

ABTS	2,2′-azino-bis-(3-ethylbenzothiazoline-6-sulfonic acid)
AC	Available Carbohydrates
AI	Atherogenicity Index
DPPH	2,2-Diphenyl-1-Picrylhydrazyl
DSC	Differential Scanning Calorimetry
FA	Fatty Acid
FAMEs	Fatty Acid Methyl Esters
FBC	Faba Bean Concentrate
FBF	Faba Bean Flour
FID	Flame Ionization Detector
FRAP	Ferric Ion Reducing Antioxidant Power
FTIR	Fourier Transform Infrared
h/H	Hypocholesterolemic/Hypercholesterolemic
OAC	Oil Absorption Capacity
SRC	Retention Capacity of Different Solvents
TDF	Total Dietary Fiber
TI	Thrombogenicity Index
WHC	Water-Holding Capacity
ΔH	Enthalpy change
